# Toll-like receptor 9 (-1237 T/C, -1486 T/C) and the risk of gastric cancer: a meta-analysis of genetic association studies

**DOI:** 10.1186/s12885-023-11509-7

**Published:** 2023-10-24

**Authors:** Yap Zi Qyi, Htar Htar Aung, Saint-Nway Aye, Wong Siew Tung, Cho Naing

**Affiliations:** 1 School of Medicine, International Medical University, Kuala Lumpur, Malaysia; 2 School of Humanities, Social Sciences and Law, University of Dundee, Dundee, Scotland, UK; 3 Faculty of Tropical Health and Medicine, James Cook University, Queensland, Australia

**Keywords:** TLR9 gene polymorphisms, Gastric cancer, Genetic association, Meta-analysis

## Abstract

**Background:**

Gastric cancer has a complex aetiology including genetic factors. Individual case-control studies of toll like receptor (TLR) 9 (-1237 T/C, -1486 T/C) polymorphisms in the gastric cancer risk were available, and they showed variation in the findings. Therefore, we performed a meta-analysis to synthesize the evidence on the association between polymorphisms of TLR 9 (-1237 T/C, -1486 T/C) and the risk of gastric cancer using data from eligible studies.

**Methods:**

This study followed the PRISMA 2020 Checklist. Studies were searched in health-related databases. The methodological quality of studies was evaluated with the use of Newcastle-Ottawa Scale criteria. The summary odds ratio (OR) and its 95% confidence interval (CI) were used to determine the strength of association between each polymorphism and the risk of gastric cancer using five genetic models. Stratification was done by ethnic groups. For the robustness of the analysis, a leave-one-out meta-analysis was performed.

**Results:**

Eight case-control studies with 3,644 participants (1914 cases, 1730 controls) were conducted across six countries. Half of the studies were conducted in China. In the NOS methodological quality assessment, only three studies received a high-quality rating (i.e., a score of ≥ 7). TLR 9 (-1486 T/C) polymorphism and the risk of gastric cancer were assessed in six studies, four of Asian ethnicity and two of non-Asian. Under the dominant model, only in the Asian ethnic group showed a marginally and significantly increased risk of gastric cancer (overall: OR = 1.22, 95%CI = 0.90–1.67, *I*^2^ = 56%; Asian: OR = 1.24, 95%CI = 1.00-1.54, *I*^2^ = 0%, non-Asian: OR = 1.25, 95%CI = 0.38–4.09, *I*^2^ = 89%). Under the recessive model in the absence of heterogeneity, only the Asian group had a significantly higher risk of developing gastric cancer (overall: OR = 1.4, 95% CI = 0.74–2.64, *I*^*2*^ = 85%; Asian: OR: 1.41, 95% CI = 1.07–1.86, *I*^2^ = 0%, non-Asian: OR = 1.18, 95% CI = 0.12–11.76, *I*^2^ = 97%). Under the heterozygous model, there was no significant association with the risk of gastric cancer overall or among any ethnic subgroup. Under the homozygous model in the absence of heterogeneity, only the Asian group had a significantly higher risk of gastric cancer (overall, OR = 1.47, 95% CI = 0.76–2.86, *I*^2^ = 82%; Asian: OR = 1.54, 95% CI = 1.13–2.1, *I*^2^ = 0%; non-Asian: OR = 1.19, 95% CI = 0.1-14.33, *I*^2^ = 96%). Under the allele model, a significantly increased risk of gastric cancer was observed only in the Asian group (overall: OR = 1.23, 95% CI = 0.89–1.71, *I*^2^ = 84%; Asian: OR = 1.22, 95% CI = 1.05–1.41, *I*^2^ = 0%; non-Asian: OR = 1.24, 95% CI = 0.34–4.59, *I*^2^ = 97%). Four studies investigated the association between TLR 9 (-1237 T/C) polymorphism and the risk of developing gastric cancer. Under any of the five genetic models, there was no association between TLR 9 (-1237 T/C) and the development of gastric cancer in overall or in any ethnic subgroup. Sensitivity analysis revealed that the effect was unstable. With a small number of studies with a small number of participants, we addressed the issue of insufficient power for drawing conclusions.

**Conclusions:**

The findings suggested that TLR9 (-1486 T/C) may play a role in the risk of gastric cancer specific to the Asian ethnic group. To substantiate the findings on the association between these two polymorphisms (TLR9 -1237 T/C, -1486 T/C) and the risk of gastric cancer, future well-designed case-control studies with a sufficient number of participants in multi-ethnic groups are recommended.

**Supplementary Information:**

The online version contains supplementary material available at 10.1186/s12885-023-11509-7.

## Background

Gastric cancer is the fourth most common cause of cancer-related death and the fifth most prevalent malignant cancer worldwide. Although declining rates, it is predicted that the global burden of stomach cancer would increase by 62% by 2040 [1]. It has been established that gastric cancer has a complex aetiology. Numerous studies have identified host-related factors, environmental factors, and *Helicobacter pylori* (*H. pylori*) colonization as risk factors for the development of gastric cancer, which modify the nature and extent of gastrointestinal disorders [2, 3]. Only 1–2% of *H. pylori*-positive patients developed distal gastric cancer, despite the fact that H. pylori infection may be the cause of a variety of gastrointestinal disorders [3]. As such, there may be a wide range of host genetic factors which affect an individual’s susceptibility to H. pylori infection. Studies reported that host genetic and epigenetic alterations could result in oncogenic overactivation and tumor suppressor pathways inactivation, leading to gastric carcinogenesis [4, 5].

Innate immunity serves as the first line of defence for human cells against foreign agents. A general differentiation between self and microbial non-self may be drawn by this innate immunity [6]. As a result, the innate immunity that exists in the stomach mucosa is crucial for the development of adaptive immune responses against *H. pylori* [7]. A family of pattern recognition receptors with a long evolutionary history is the toll-like receptors (TLRs). They were the first family of proteins, in fact, to exhibit Janeway’s predictions that would characterize pattern recognition receptors (PRRs) [8]. Ten receptors are involved in the detection of specific pathogen-associated molecular patterns (PAMPs). For instance, TLR4 is activated by lipopolysaccharide [9], TLR5 is activated by flagellin [10], and TLR9 is activated by unmethylated CpG patterns of bacterial and viral DNA [11], as previously described [12, 13].

 Published Genome-Wide Association Studies (GWASs) of gastric cancer in the HuGE PubLit database and the GWAS CatLog of National Human Genome Research Institute (NHGRI) have not examined TLR 9 and its variants. We are aware of the meta-analysis that assessed TLR 2 and TLR 4 in the risk of gastric cancer [13]. Studies on TLR 9 polymorphisms and their relationship with gastric cancer risk emerged in relation to its potentials for initiating an immune response in the presence of *H. pylori* [14]. The results from these studies are inconsistent. Individual studies that assessed the link between TLR 9 and gastric cancer risk featured a variation in sample size, geographical locations, and ethnic groups, among others. Meta-analysis is a quantitative approach for combining results from various studies on the same topic, and for estimating and explaining their diversity [15, 16]. It is growing as a popular method for resolving discrepancies in genetic association studies [17]. Meta-analysis of genetic association studies is regarded as decisive evidence when carried out properly [18]. We are aware of a meta-analysis with TLR 9 and the cancer risks, where only two individual case-control studies with gastric cancer were included [19]. More individual case-control studies of TLR 9 (-1237 T/C, -1486 T/C) polymorphisms in the gastric cancer risk were available after releasing the Zhang review [19]. Taken together, the objective of this meta-analysis was to synthesize the evidence on the association between polymorphisms of TLR 9 (-1237 T/C, -1486 T/C) and the risk of gastric cancer using data from eligible studies. The finding will help decision makers to identify a high risk group for screening for gastric cancer.

## Methods

We followed PRISMA 2020 standards for the reporting of this review (Additional file 1). This meta-analysis study was approved by the Ethics Review Committee of the International Medical University (IMU) in Malaysia (ID: IMU I/BMS1/2021(02). A protocol of the current meta-analysis is available from the corresponding author on reasonable request. This study solely used published data, and therefore the need for consent from participants was waived by the Institutional Ethics Review Committee.

### Study selection

We searched relevant studies in electronic databases of Medline via the PubMed interface, EMBASE, EBSCOHOST, Science Direct, and Google scholar. The search terms were “toll-like receptor 9”, “toll 9 receptor” “TLR9” “TLR 9 rs5743836”, “TLR 9 rs187084, “TLR 9” “TLR 9 -1237”, “TLR 9 -1486”, “gastric cancer”, with an appropriate Boolen operator (AND/ OR). The last date of search was December 2022. We also applied the snowball method using manual cross-referencing from retrieved articles to ensure a comprehensive search. Single nucleotide polymorphism identification numbers (SNPs) (rs numbers) were also applied for identification of the eligible studies. Details of the MeSH and terms used are presented in Additional file 2.

### Selection criteria

Individual studies were selected based on the following criteria: (1) human study of case- control design, (2) inclusion of cases with confirmed gastric cancer, and controls of gastric cancer -free participants, (3) sufficient information on frequencies of SNPs in both cases and controls − 1237 T/C (rs5743836, rs574383), -1468 T/C (rs352139, rs187084, rs41308230, rs5743844), and (4) use of DNA-based method for genotyping.

To be eligible, a study must provide sufficient information to extract (calculate) an odds ratio (OR) and its 95% confidence intervals (CI). Studies were not considered if they did not meet the inclusion criteria. Hence, non-empirical studies such as editorials, letters to editors, and methodological studies were not considered. Studies of descriptive case series, clinical interventions, preclinical studies or epidemiologic studies such as risk factor analysis of gastric cancer were also excluded.

### Data extraction

Two investigators (YZQ and HHA) independently selected the included studies through a four-phase selection process as displayed in the PRISMA-2020 flowchart. The two investigators independently collected data from each study with a use of piloted data extraction sheets. Collected data were: first author, year of study, country of study, tumour location (cardia, non-cardia), ethnicity, SNP types, detection methods, the total number of cases and controls, frequencies of alleles and genotypic distributions in cases and controls, status of Hardy-Weinberg equilibrium (HWE), and minor allele frequency (MAF). If MAF was not provided in the study, we derived it. If HWE was not provided in the study, it was assessed in the controls using a goodness of fit chi-square test; a *p* value less than 0.05 was deemed significant disequilibrium [20]. Any disagreements in these steps were resolved by discussion with the third investigator (SNA).

### Quality assessment

The methodological quality of the studies was evaluated with the Newcastle-Ottawa Scale (NOS) criteria [21]. This tool evaluates three main domains: the selection and representativeness of cases and controls, comparability of cases and controls, and the methods of ascertainment for cases and controls. The NOS criteria’s quality score ranges from zero to nine, in which a higher score represents a better-quality study. Two reviewers (YZQ and HHA) assessed a quality score for each item. Any disagreement between the two investigators was resolved by discussion with the third investigator (SNA).

### Statistical analysis

For each study identified, study-specific variance of the natural logarithm of adjusted OR and its 95% CI were derived to measure the risk effect of gastric cancer and TLR 9 (-1237 T/ C, -1486 T/ C). For the overall pooled analysis (i.e., the main analysis of this meta-analysis), we estimated the summary OR and its 95%CI of all included studies stratified by ethnic groups, regardless of their HWE status. Summary estimates were obtained using the random-effects models (i.e. DerSimonian and Laird), reflecting substantial between-study heterogeneity [22]. Otherwise, a fixed effect model would be used. Between-studies heterogeneity was assessed with Cochran’s *Chi*^2^ -based Q statistic (*P value for Q statistic* < 0.10 is considered significant heterogeneity) [23], and the *I*^2^ statistics [24]. The *I*^2^ represents the percentage (%) of the observed between-study variability due to heterogeneity rather than to chance. It ranges between 0% and 100%; values above 75% imply substantial heterogeneity [24]. Meta-analysis was performed with five genetic models such as allelic contrast, dominant, recessive, homozygous, and heterozygous models to determine the association between TLR 9 (-1486 T/C, -1237 T/C) and gastric cancer risk. Analysis was stratified by two broad ethnicities such as Asian and non-Asians. The category ‘Asian’ includes all individuals who identify with one or more nationalities or ethnic groups originating in the Far East, Southeast Asia, or the Indian subcontinent [25]. It reflects a geographic location-based category, rather than biological functioning categories. Non-Asians in this study cover those participants other than Asian countries. For robustness of estimates, sensitivity analyses were done with a leave-one out meta-analysis.

A minimum of ten studies is required to conduct Egger’s test for publication bias [ 25]. Publication bias was not done due to the presence of eight studies only. Data analysis was done with RevMan 5.4 (Copenhagen), and *meta* package of R (version 4.3).

## Results

**Fig. 1:**
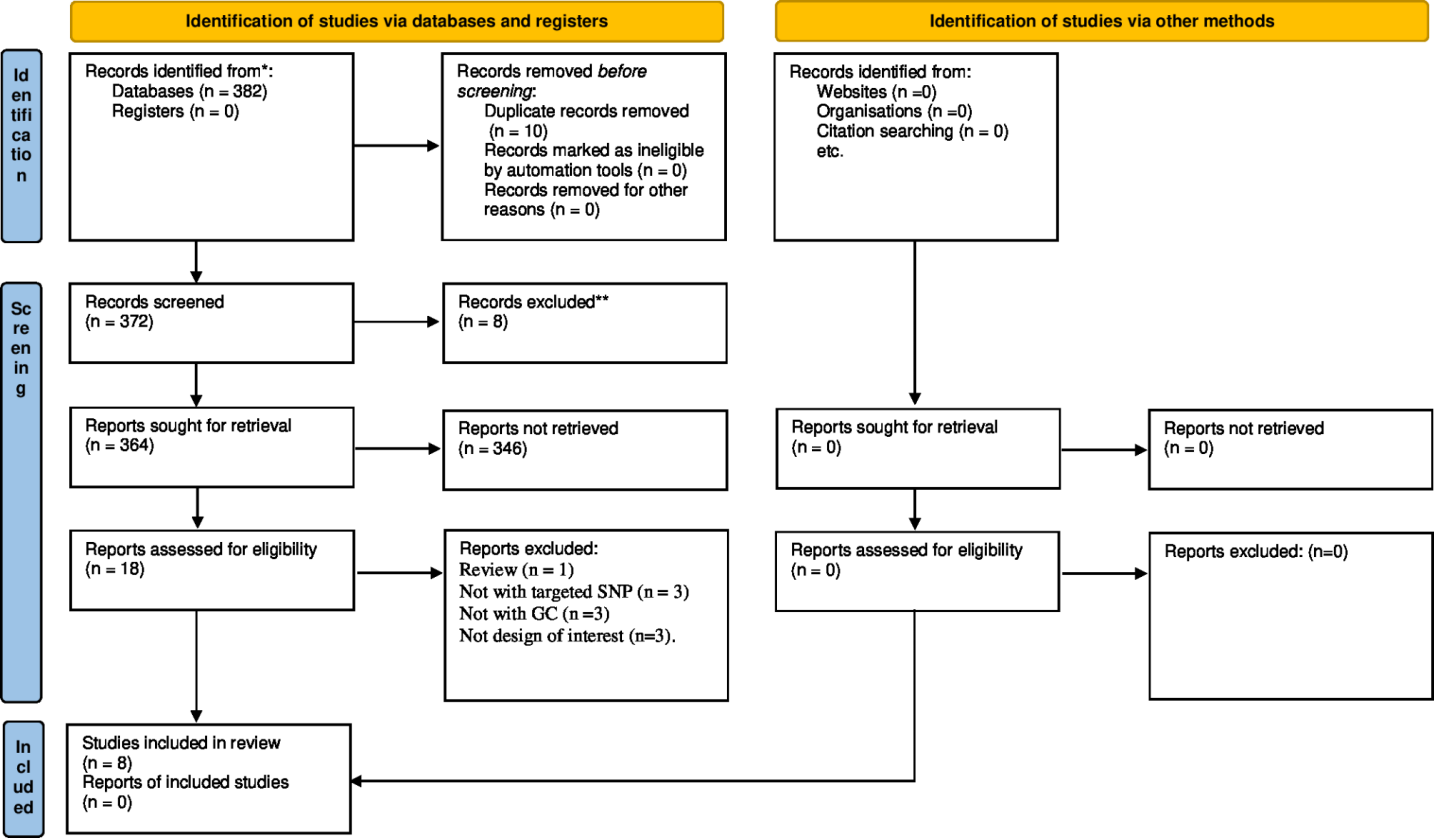
Study selection process

Figure 1 presents the study selection process. The initial search yielded 382 studies from the databases and one additional study via manual searching. Ten duplicate studies were then removed. After screening the titles and abstracts, 346 studies were further excluded. Eighteen full-text studies were checked, and ten studies were excluded for different reasons. A final of eight studies that assessed the association of TLR 9 polymorphisms (-1486 T/C, -1237 T/C) were eligible for this meta-analysis [5, 14, 26–31]. The excluded ten studies and their justifications were provided in Additional file 3.

### Study characteristics and quality assessment

Eight studies (nine datasets), incorporating 3,644 participants (1,914 cases, 1,730 controls) across nine countries that assessed the association of two TLR 9 polymorphisms (-1486 T/C, -1237 T/C) were eligible for the current analysis. The main characteristics of the eight included studies is provided in Table 1. One study (two datasets) assessed TLR 9 (-1237 T/C) [14], five studies assessed TLR 9 (-1486 T/C) [5, 26, 29–31], and two studies assessed both SNPs (-1237 T/C and − 1496 T/C) [27, 28]. Half of the studies were conducted in a single country in the Asian region such as China [5, 26, 29, 30], while four studies in the non-Asian countries such as Brazil [27], Egypt [31], Italy [28], and Poland as well as the USA [14]. These eight studies were published between 2009 and 2022. Seven studies were hospital-based case-control designs [5, 26–31], and only one was a population-based study [14]. Half of these studies used PCR-RFLP [5, 26, 30, 31], and another half used TaqMan for genotyping [14, 27–29]. For TLR 9 (-1237 T/C), only one study [27] violated HWE in the control participants. This happened in two studies of TLR 9 (-1486 T/C) [5, 31]. The NOS methodological quality assessment rated the included studies ranged from six to eight stars (Additional file 4), in which only three studies were of high quality (i.e., ≥ 7) [26, 29, 31]. The genotype frequencies of each study identified in the current meta-analysis are provided in Table 2. The distribution of MAF among the studies ranged from 9 to 42.5%, reflecting that none was ‘monomorphic’ [32] and all were ‘common’ distribution.

**Table 1:** Characteristics of the included studies

Study, yr	Country	Setting	cases/ controls	Type of cases/ controls	Mean age yr (± SD) in cases	Male% in cases	SNP	Method	HWE	GC confirmations
Liu, 2015 [5]	China	HB	209/94	GC/healthy	59.2 (11.1)/ 55.7 (17.3)	53.11/52.13	-1486 T/C	PCR-RFLP	No	histo
Hold 2009 [14]	Poland	PB	327/406	GC/healthy	≥50: 89.4%^!^	65%^3^	-1237 T/C	TaqMan	Y	intestinal pathological type^!^
Hold 2009 [14]	USA	PB	306/211	GC/healthy	md:66^2^	85%^2^	-1237 T/C	TaqMan	Y	cardia adeno & other gastric adeno
Wang, 2013 [26]	China	HB	314/314	GC/healthy	60 (12.61)/ 59 (12.01)	67.2/67.2	-1486 T/C	PCR-RFLP	Y	histo
Susi, 2019 [27]	Brazil	HB	161/200	GC/healthy	43.10 ± 21.79	44%^3^	both	TaqMan	Y (-1486 T/C) ; No (-1237 T/C)	histo
Valli, 2019 [28]	Italy	HB	114/97	GC/ healthy	61.45 (1.04)/ 42.03 (1.66)	62.3/56.7	both	TaqMan	Y	histo
Gao, 2020 [29]	China	HB	288/ 281	GC/healthy	59.5 (11.2)/ 59.1 (11.57)	77.8/78.3	-1486 T/C	TaqMan	Y	histo; non-cardia
Ding, 2022 [34]	China	HB	63/22	GC/healthy	NA	NA	-1486 T/C	PCR-RFLP	Y	biopsies; gastric atrophy/intestinal metaplasia
Sultan, 2022 [31]	Egypt	HB	106/106	GC with + ve HP/healthy HP negative	56.55 (8.63)/ 53.27(8.55)	59.4/55.7	-1486 T/C	PCR- RFLP	No	histo

^1^: adapted from Chow WH, et al. Risk of stomach cancer in relation to consumption of cigarettes, alcohol, tea and coffee In Warsaw, Poland. Int J Cancer 1999:871–6

^2^: adapted from Gammon MD, et al. Tobacco, alcohol, and socioeconomic status and adenocarcinomas of the esophagus and gastric cardia. J Natl Cancer Inst 1997; 89:1277–84

3: percentage of total males in the study

Adeno: adenocarcinoma: Both; both SNPs (-1237 T/C, -1486 T/C); GC: gastric cancer; HB: hospital-based data collection; histo: histopathological confirmation; HWE: Hardy-Weinberg equilibrium; Md: median; Method: Genotyping method; NA: not available; PB: Population-based data collection; PCR-RFLP: Polymerase chain reaction-restriction fragment length polymorphism; SD: standard deviation; SNP: Single nucleotide polymorphisms; Y: Yes, HWE satisfied; Yr: year(s)

**Table 2:** Distribution of genotype frequencies of included studies

SNP	Study, yr	Country	Total cases	Cases					Total controls	Controls						
				TT	TC	CC	T	C		TT	TC	CC	T	C	HWE	MAF
-1237 T/C	Ding 2022 [34]	China	63	36	27	0	99	27	21	17	4	0	38	4	0.63	0.095
	Hold 2009 [14]	Poland	326	261	58	7	580	72	406	316	85	5	717	95	0.79	0.116
	Hold 2009 [14]	USA	298	224	69	5	517	79	210	149	57	4	355	65	0.58	0.154
	Susi, 2019 [27]	Brazil	161	78	44	39	200	122	200	148	40	12	336	64	0.00	0.16
	Valli, 2019 [28]	Italy	114	80	30	4	190	38	97	70	25	2	165	29	0.89	0.149
-1486 T/C	Liu, 2015 [5]	Chinese	209	58	111	40	227	191	94	29	55	10	113	75	0.03	0.399
	Wang, 2013 [26]	China	314	94	164	56	452	276	314	122	148	44	392	236	0.93	0.399
	Ding, 2022 [34]	China	63	18	31	14	67	59	22	8	11	3	27	17	0.80	0.386
	Gao, 2020 [29]	China	286	96	134	56	326	246	272	93	136	43	322	222	0.56	0.408
	Sultan, 2022 [31]	Egypt	106	22	28	56	78	140	106	40	42	24	122	99	0.052	0.425

HWE: Hardy-Weinberg equilibrium; MAF: minor allele frequency [(C alley x2)/ (T allele + C allele) x 2]

### TLR 9 (-1486 T/C) and the risk of gastric cancer

**Fig. 2:**
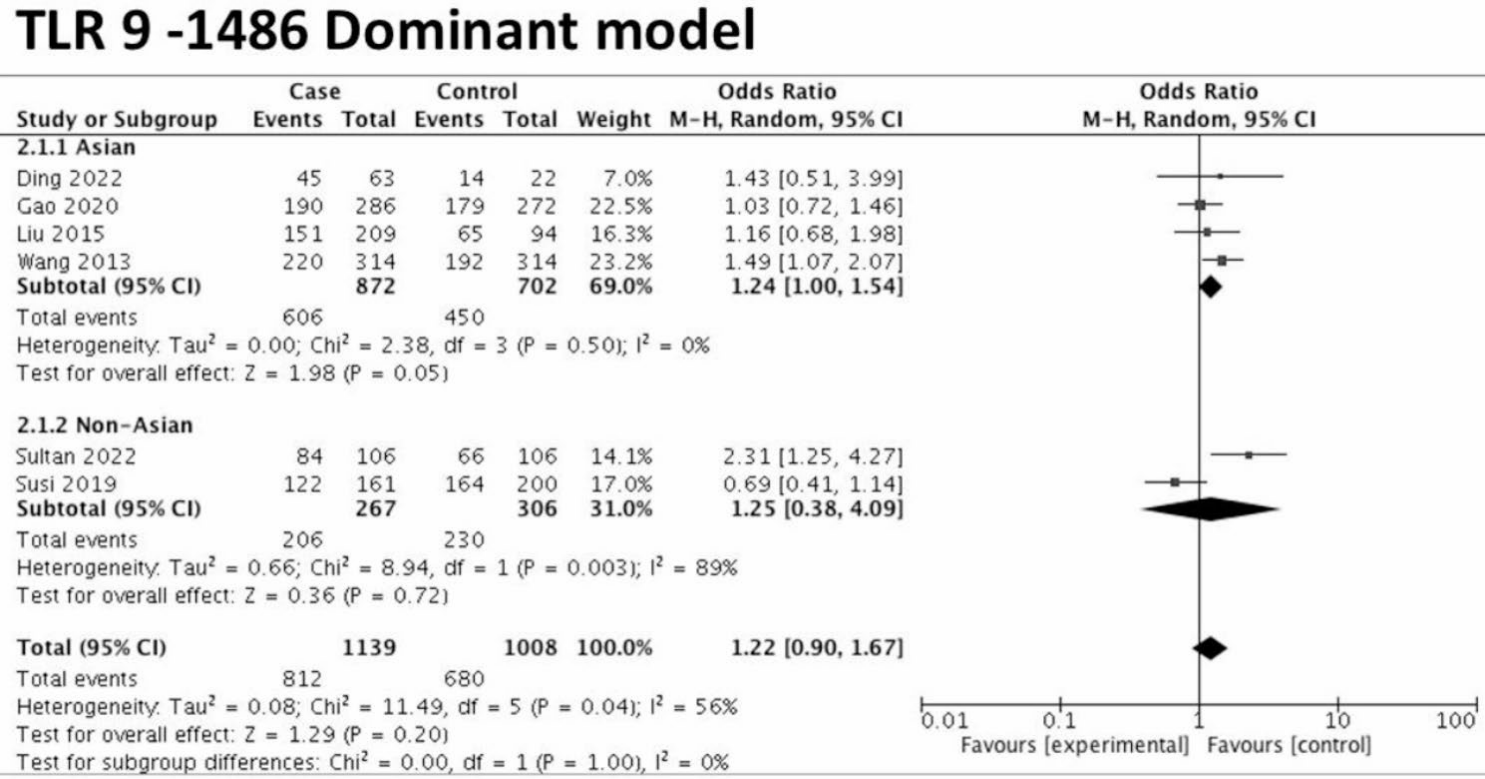
Forest plot showing the effect estimates for TLR 9-1486 (T/C)

Overall, six studies (i.e., Asian ethnic group: 4 studies, non-Asian ethnic group: 2 studies) investigated the association between polymorphism of TLR 9 (-1486 T/C) and the gastric cancer risk [5, 26, 27, 29–31]. All except one study [33] satisfied the HWE. Under the dominant model, it was marginally and significantly associated with the risk of gastric cancer only in the Asian ethnic subgroup (overall: OR = 1.22, 95%CI = 0.90–1.67, *I*^*2*^ = 56%; Asian: OR = 1.24, 95%CI = 1.00–1.54, *I*^*2*^ = 0%, non-Asian: OR = 1.25, 95%CI = 0.38–4.09, *I*^*2*^ = 89%) (Fig. 2). Under the recessive model, there was a significantly increased risk of gastric cancer only in the Asian group (overall: OR = 1.4, 95%C I = 0.74–2.64, *I*^*2*^ = 85%; Asian: OR: 1.41, 95% CI = 1.07–1.86, *I*^*2*^ = 0%, non-Asian: OR = 1.18, 95%CI = 0.12–11.76, *I*^*2*^ = 97%) (Additional file 5). Under the heterozygous model, there was no significant association with the risk of gastric cancer in overall or any ethnic subgroup (overall: OR = 1.12, 95% CI = 0.92–1.36, *I*^2^ = 0%; Asian: OR = 1.16, 95% CI = 0.92–1.45, *I*^*2*^ = 0%; non-Asian: OR = 0.99, 95% CI = 0.65–1.51, *I*^2^ = 0%). Under the homozygous model, a significantly increased the risk of gastric cancer was observed only in the Asian group (overall, OR = 1.47, 95% CI = 0.76–2.86, *I*^2^ = 82%; Asian: OR = 1.54, 95% CI = 1.13–2.1, *I*^*2*^ = 0%; non-Asian: OR = 1.19, 95% CI = 0.1-14.33, *I*^2 =^ 96%) (Additional file 5). Under the Allele model, a significantly increased the risk of gastric cancer was observed only in the Asian group (overall: OR = 1.23, 95% CI = 0.89–1.71, *I*^*2*^ = 84%; Asian: OR = 1.22, 95% CI = 1.05–1.41, *I*^2^ = 0%; non-Asian: OR = 1.24, 95% CI = 0.34–4.59, *I*^2^ = 97%) (Additional file 5).

### TLR 9 (-1237 T/C) and the risk of gastric cancer

**Fig. 3:**
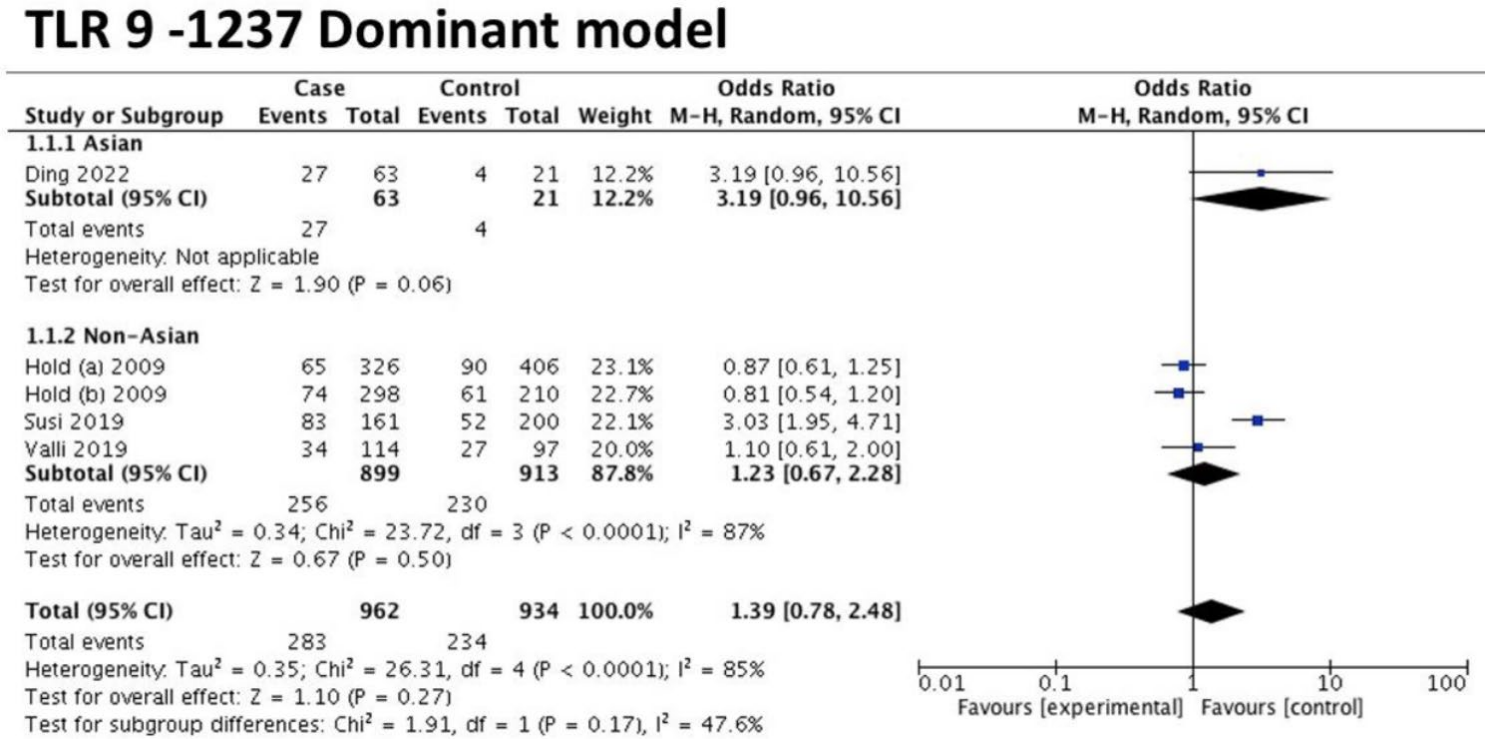
Forest plot showing the effect estimates for TLR 9-1237 (T/C)

Overall, four studies with five datasets (i.e., Asian ethnic group: 1 study, non-Asian ethnic group: 3 studies) assessed the association between this polymorphism and the gastric cancer risk [14, 27, 28, 30]. Under the dominant model, there was no significant association between TLR 9 (-1237 T/C) and the risk of gastric cancer in overall or any ethnic subgroups (overall: OR = 1.39, 95%CI = 0.78–2.48, *I*^*2*^= 85%; Asian: OR = 3.19, 95%CI = 0.96–10.56, *I*^*2*^ = 0%, non-Asian: OR = 1.23, 95%CI = 0.67–2.28, *I*^*2*^ = 87%) (Fig. 3). So were the remaining four genetic models (Additional file 6) and (Table 3).

**Table 3:** The effect estimates for TLR 9-1486 T/C and − 1237 T/C

SNP (Total number of studies)	Genetic Model	Effect estimates, OR, (95%CI)		
TLR 9 -1237 T/C (4 studies)		Overall	Asian group	Non-Asian group
	Dominant	1.39 (0.78, 2.48) ^1^	not estimable^2^	1.23 (0.67, 2.28)
	Recessive	2.20 (0.93, 5.18)	not estimable^2^	2.20 (0.93, 5.18)
	Homozygous	2.21 (0.81, 6.04)	not estimable^2^	2.21 (0.81, 6.04)
	Heterozygous	1.20 (0.77, 1.86)	not estimable^2^	1.08 (0.70, 1.65)
	Allele	1.42 (0.78, 2.59)	not estimable^2^	1.30 (0.68, 2.50)
TLR 9 -1486T/C (6 studies)	Dominant	1.22 (0.90, 1.67)	**1.24 (1.00, 1.54)**	1.25 (0.38, 4.09)
	Recessive	1.40 (0.74, 2.64)	**1.41 (1.07, 1.86)**	1.18 (0.12, 11.76)
	Homozygous	1.47 (0.76, 2.86)	**1.54 (1.13, 2.10)**	1.19 (0.10, 14.33)
	Heterozygous	1.12 (0.92, 1.36)	1.16 (0.92, 1.45)	0.99 (0.65, 1.51)
	Allele	1.23 (0.89, 1.71)	**1.22 (1.05, 1.41)**	1.24 (0.34, 4.59)

^1^Inclusive of one study with the Asian ethnic group; ^2^ Only one study identified for the Asian ethnic group. Bold denotes statistical significance. CI: confidence interval; OR: odds ratio

### Sensitivity analyses

We conducted a leave-one-out sensitivity analysis by sequentially discarding one study at a time due to variations in sample size, genotyping techniques, and the presence of small studies. The results of the sensitivity analysis revealed that the effect estimates were unstable. For instance, TLR 9 (-1237 T/C) and the risk of gastric cancer among non-Asians and the overall group were significantly related under the allele model (i.e., after omitting a study [30]). None had been determined to be significantly associated in the initial analysis. This demonstrated that the estimates were unstable. In the recessive, homozygous, and allele models (i.e. after omission), TLR 9 (-1486 T/C) and the risk of gastric cancer was significantly related in Asians, non-Asians, and the whole population. This was different from the findings in the initial analyses (Table 4), reflecting the instability of the estimates.

The estimates for all five genetic models were reanalysed after a study that departed from HWE in TL9 (-1486T/C) [31] was removed, and the different findings were shown. As an example, under the recessive model, significant associations have been observed in both Asian and non-Asian groups with different directions (overall, OR = 1.06; 95%CI = 0.83–1.35; Asian: OR: 1.42; 95%CI = 1.08–1.47; non-Asian: OR = 0.37; 95%CI = 0.21–0.64). The same was true for the other four genetic models (Table 4). This implied HWE deviation also had an impact on the effect estimates.

**Table 4:** The estimates of a leave-one- out meta-analysis

SNP, genetic model	Study excluded [ref]	OR, (95% CI)		
		Overall	Asian group	Non-Asian group
TLR 9 (-1237 T/C)		4 studies	1 study	3 studies
allele	Ding, 2022 [30]	**1.33, 1.11–1.59**	Not estimable	**1.33, 1.11–1.59**
TLR 9 (-1486 T/C)		6 studies	4 studies	2 studies
dominant	Susi, 2019 [27]	**1.33, 1.09–1.63**	**1.24, 1.00–1.54**	**2.31, 1.25–4.27**
	Sultan, 2022. [31]	1.14, 0.93–1.38	**1.24, 1.00–1.54**	0.69, 0.41–1.14
recessive	Susi, 2019 [27]	**1.70, 1.33–2.18**	**1.42, 1.08–1.87**	**3.83, 2.11–6.93**
	Sultan, 2022 [31]	1.06, 0.83–1.35	**1.42, 1.08–1.47**	**0.37, 0.21–0.64**
homozygous	Susi, 2019 [27]	**1.82, 1.37–2.40**	**1.54, 1.13–2.10**	**4.24, 2.09–8.60**
	Sultan, 2022 [31]	1.17, 0.89–1.54	**1.54, 1.13–2.10**	**0.33, 0.17–0.66**
allele	Susi, 2019 [27]	**1.33, 1.16–1.52**	**1.22, 1.05–1.41**	**2.43, 1.65–3.59**
	Sultan, 2022 [31]	1.07, 0.94–1.22	**1.22, 1.05–1.41**	**0.64, 0.48–0.86**
homozygous	Susi, 2019 [27]	**0.55, 0.42–0.73**	**0.65, 0.48–0.88**	**0.24, 0.12–0.48**
	Sultan, 2022 [31]	0.85, 0.65–1.12	**0.65, 0.48–0.88**	**2.99, 1.52–5.87**
TLR 9 -1486 T/C	Susi, 2019 [27]	**0.75, 0.66–0.86**	**0.82, 0.71–0.95**	**0.41, 0.28–0.61**
	Sultan, 2022 [31]	0.87, 0.76–1.00	**0.82, 0.71–0.95**	**2.99, 1.52–5.87**

Bold denotes statistical significance; OR: odds ratio

## Discussion

Given the association between inflammation and carcinogenesis, candidate gene approaches are becoming more and more attractive for identifying genes that may initiate and progress inflammation-associated carcinogenesis, particularly gastric cancer, in the gastrointestinal tract. In this meta-analysis, we evaluated the influence of two prominent SNPs (1237 C/T and − 1486 T/C) in the TLR9 gene on the risk of gastric cancer across six countries.

A previous review that included two case-control studies on gastric cancer revealed a lack of statistical significance between TLR 9 (-1237 T/C) and the risk of gastric cancer employing the selected inheritance model [15]. This is comparable to our analysis of the four studies that satisfied the HWE. This means that independent of the included studies and total samples, TLR 9 (-1237 T/C) has no functional role in the development of gastric cancer. In our study, there were no reliable estimates of the association with TLR9 (-1486 T/C). In general, the results that are not significant may be attributed to (1) the inability to adjust the conventional risk factors, such as a family history of gastric cancer, or common risk factor such as *H. Pylori* infection [2] a lack of power to detect a significant association as a consequence of a paucity of studies, and [3] the within-population heterogeneity, geographic variation, and difference in source population (i.e., hospital-based vs. population-based). It also seems that these SNPs may be more closely related to non-significant gastric cancer risk. There is also a likelihood that these SNPs may have significance for defining the host immune response to *H. pylori* infection, but it does not appear that they determine what happens subsequently in the development of cancer [14]. Notable are only one Asian study and three studies with Caucasian groups for TLR 9 (-1237 T/C), which might be confounded by population stratification.

### Study limitations

There are several limitations that should be acknowledged. First, the sample sizes in the current study were small. For example, less than ten studies were identified, and only one study included the Asian-subgroup in TLR 9 (-1237 T/C). Hence, a type II error is a concern. We acknowledge the issue that there may be more specific ethnic differences that exist amongst populations in the same country. Also, there might be a common confounding factor such as age, gender, *H. pylori* infection status. Due to the inconsistent manner of reporting, we were unable to do meta regression with common covariates. Moreover, in small studies identified by the current analysis, a statistically significant finding would actually be a false-positive report probability (FRPP). The FRPP, which is the probability of no association between a genetic variant and a disease (i.e., gastric cancer in this case) gives statistically significant results in terms of the observed *p* value, the prior probability that the association between the genetic variant and the disease (i.e., gastric cancer in this case) is real, and the statistical power of the test [32]. We included only published studies in English. Hence, there might be relevant studies in other languages or non-published studies, which could contribute to ‘information bias’. It was not possible to assess reporting bias by creating a funnel plot because there were fewer than ten trials included. The subgroups of Asians and non-Asians were location-based categories, not solely reflecting biological categories or ethnic differences. This broad classification is an ease of the current analysis, but care must be taken to interpret that the findings did not represent the actual ethnic differences in genotype distribution.

The included studies used different genotyping methods, which might be associated with different genotyping success rates and data quality. However, genotyping errors are expected to be small, and thus the resulting biases are likely to be small [33]. As the aetiology of gastric cancer is complex, the influence of polymorphisms in the TLR 9 gene might be covered by unidentified genes and environmental factors. Hence, the comprehensive analysis of gene-gene interaction and gene-environment interaction might be more informative. It is, however, beyond the scope of our analysis.

### Implications for clinical practice

Based on limited data presented in this review, we still do not confirm whether TL9 gene polymorphisms (-1237 T/C and − 1486 T/C) increase or reduce the risk of gastric cancer. Our analysis highlights the need for additional studies on the role of the TLR 9 gene polymorphisms in the risk of gastric cancer, and the findings should be used to inform healthcare providers considering screening for genetic risk factors. Such research could aid in identifying patients who have considerably higher risks of disease progression and may guide the development of customized prevention and management plans for *H. Pylori* infection.

## Conclusion

The findings suggested that TLR9 (-1237 T/C and − 1486 T/C) have some roles in the associated risk of gastric cancer. The information size was inadequate to achieve confirmatory evidence. Future well-designed case-control studies with an adequate number of participants in multi-ethnic groups, stratified with common factors, are recommended to substantiate the evidence on the relationship between these two polymorphisms and gastric cancer risk.

### Electronic supplementary material

Below is the link to the electronic supplementary material.

Supplementary Material 1

Supplementary Material 2

Supplementary Material 3

Supplementary Material 4

Supplementary Material 5

Supplementary Material 6

## Data Availability

All data generated or analysed during this study are included in this published article and its supplementary information files.

## List of abbreviations

CI: Confidence interval
GC: Gastric cancer
GWAS: Genome-Wide Association Study
FPRR: False- positive report probability
*H. pylori*: *Helicobacter pylori*
HWE: Hardy–Weinberg equilibrium
MAF: Minimum Alley frequency
NHGRI: National Human Genome Research Institute
NOS: Newcastle–Ottawa scale
OR: Odds ratio
PCR: Polymerase chain reaction
PCR/RFLP: PCR/Restriction Fragment Length Polymorphism
PRRs: Pattern recognition receptors
PRISMA: Preferred Reporting Items for Systematic reviews and Meta-Analyses
SNP: Single nucleotide polymorphisms
TLRs: Toll-like receptors
