# Supplementary figures and images for: Toll-like receptor 9 (-1237 T/C, -1486 T/C) and the risk of gastric cancer: a meta-analysis of genetic association studies
Source: BMC Cancer. 2023 Oct 24;23:1027. doi: 10.1186/s12885-023-11509-7 (PMC10594725; doi:10.1186/s12885-023-11509-7)

Additional File 2 . Search strategies


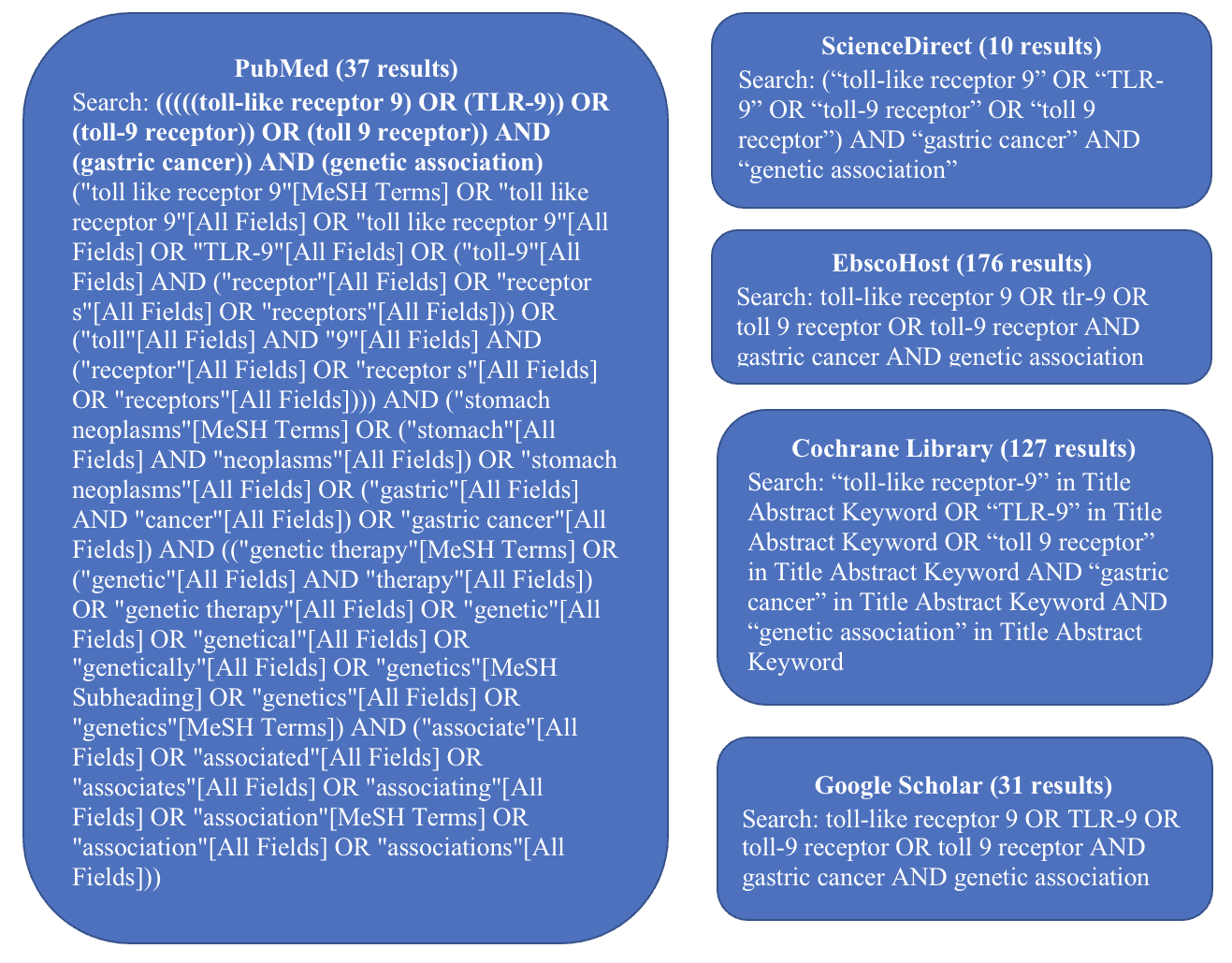

Supplement: Supplementary file 1 — Supplementary Material 1 [file 12885_2023_11509_MOESM1_ESM.doc]

Additional File 4. **Assessment of methodological quality of studies by NOS criteria**


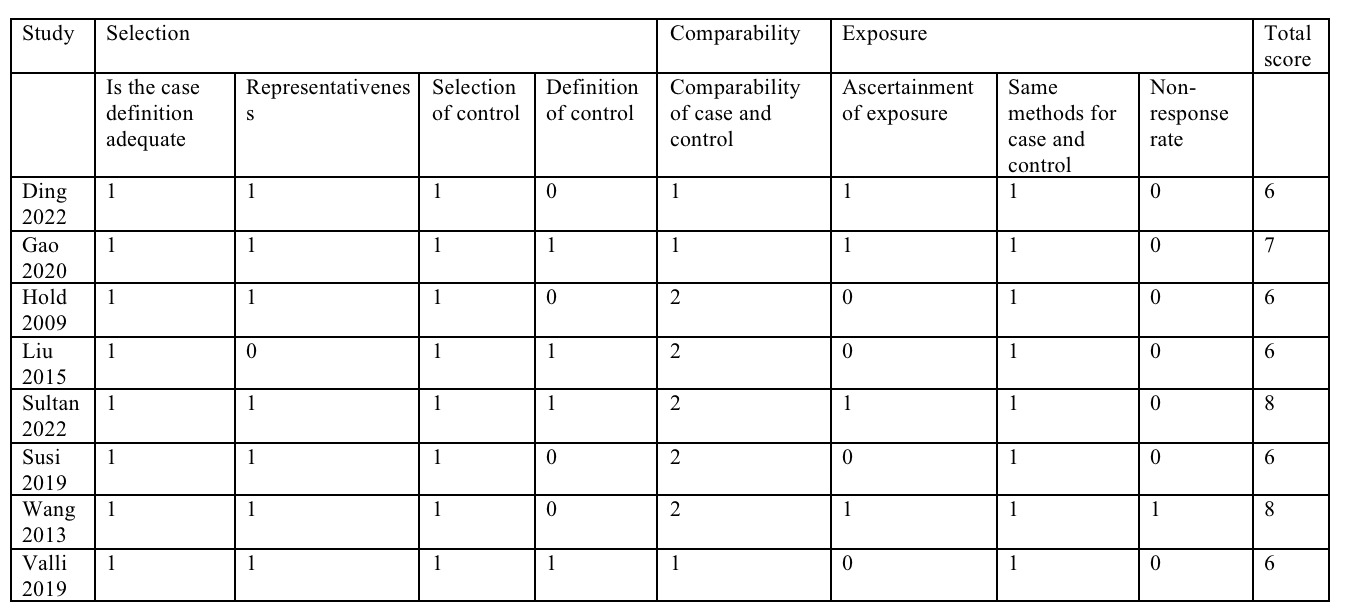

Supplement: Supplementary file 3 — Supplementary Material 3 [file 12885_2023_11509_MOESM3_ESM.doc]

**Additional File 5. Forest plot showing the effect estimates for TLR 9 (-1486 T/C)**


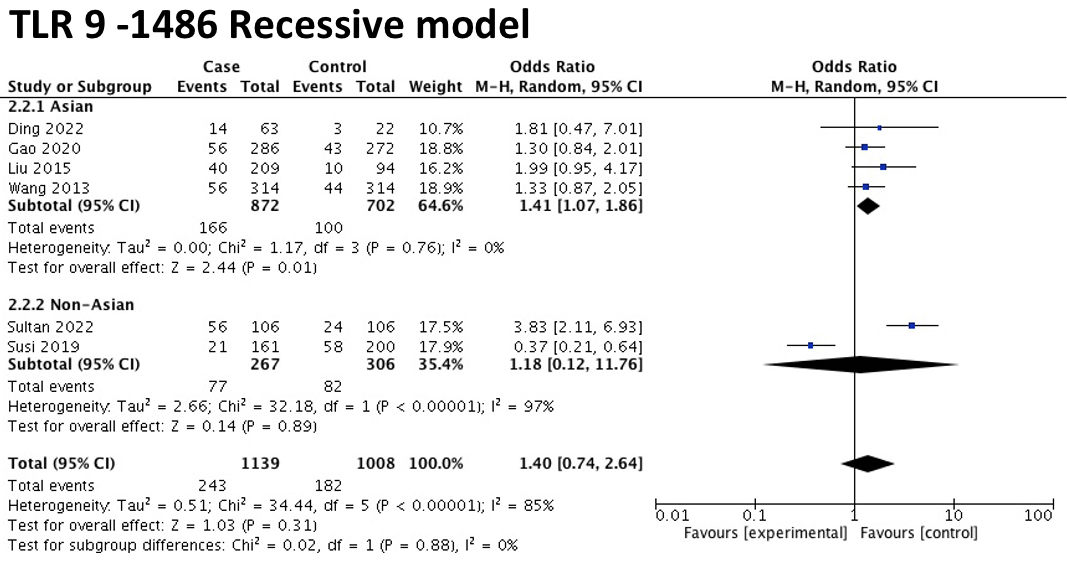


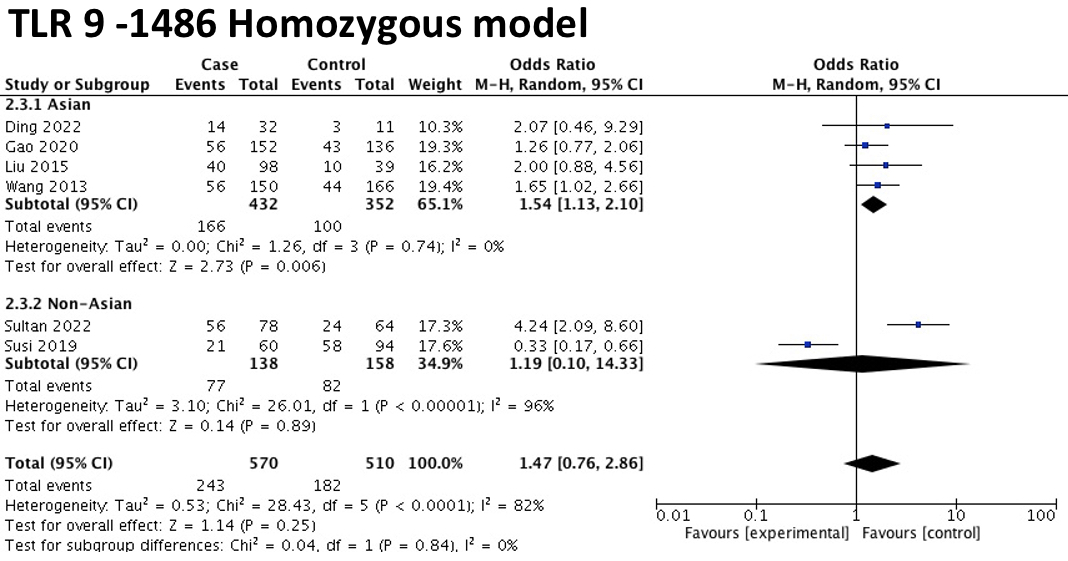


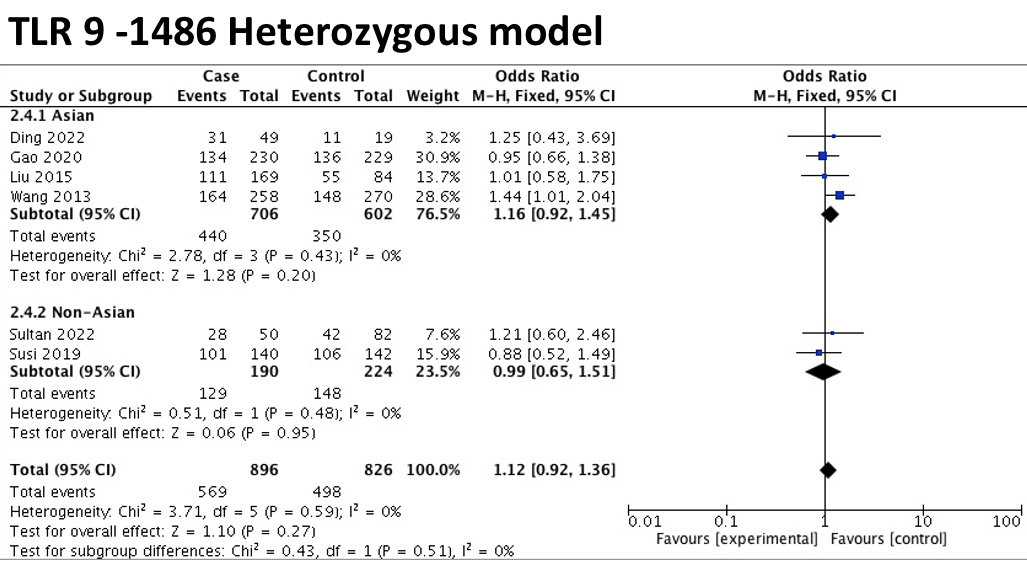


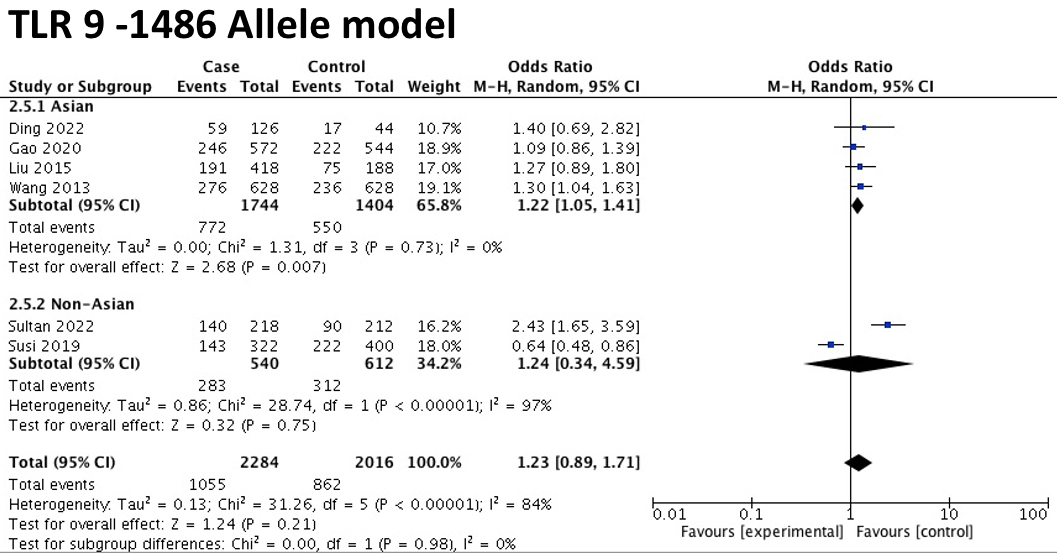

Supplement: Supplementary file 4 — Supplementary Material 4 [file 12885_2023_11509_MOESM4_ESM.doc]

**Additional File 6. Forest plot showing the effect estimates for TLR 9(-1237 T/C)**


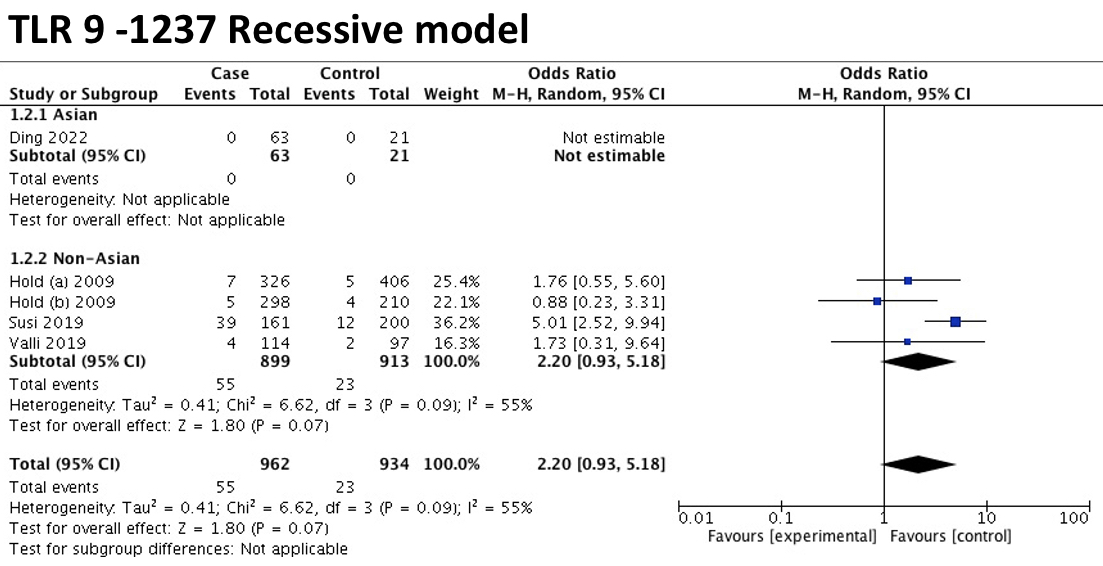


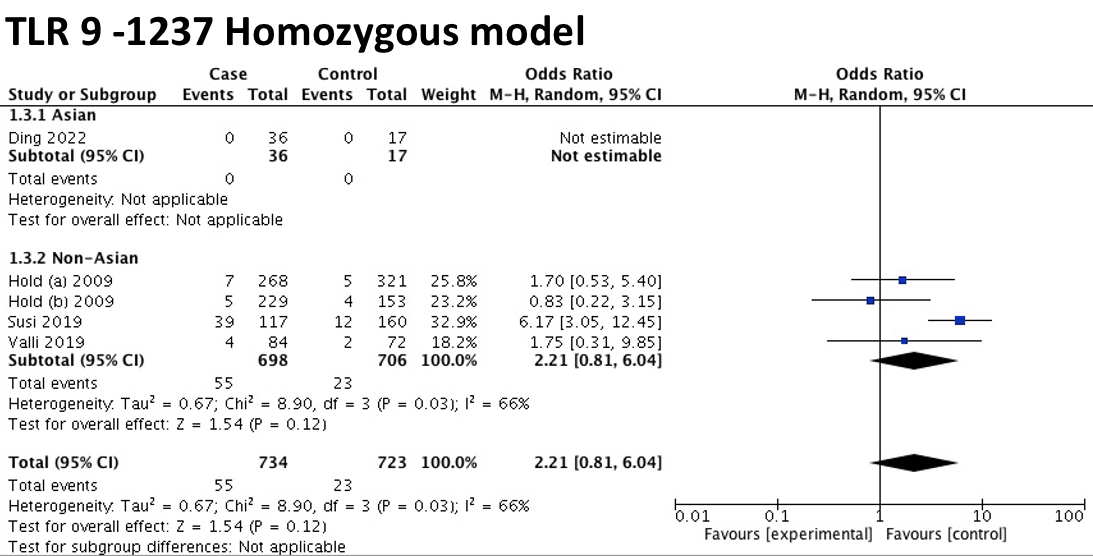


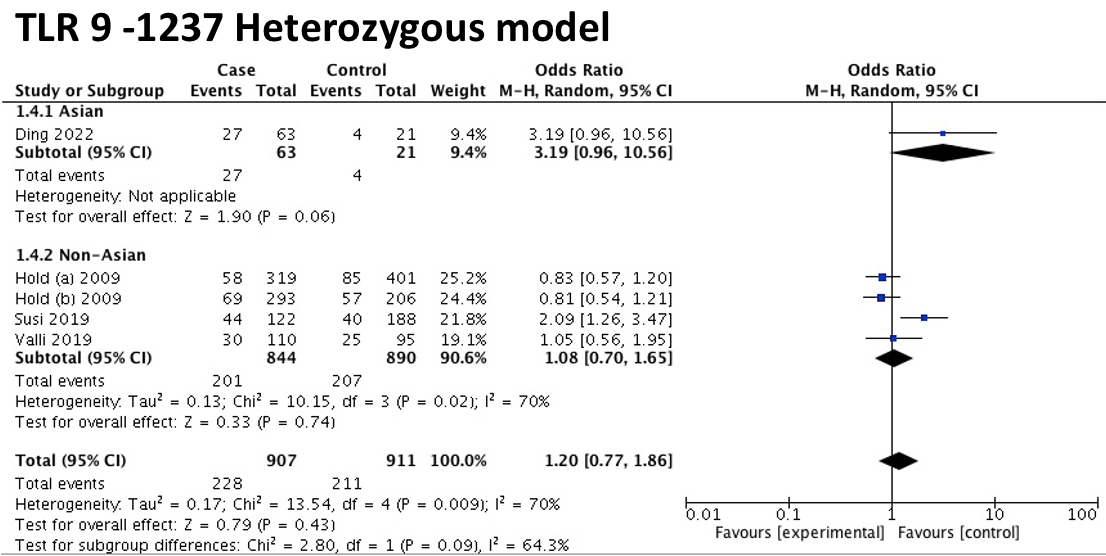


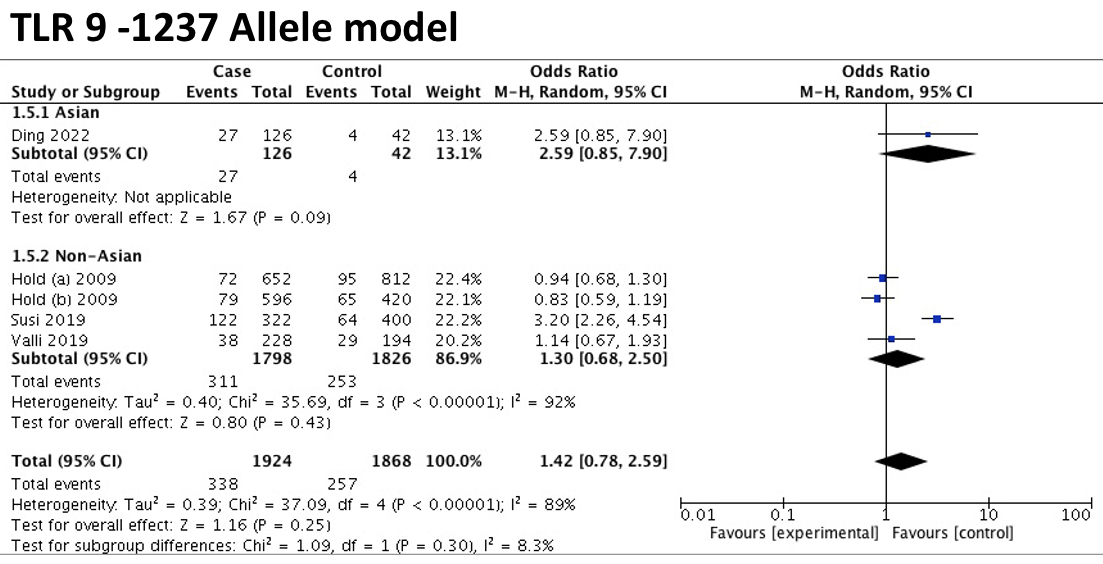

Supplement: Supplementary file 5 — Supplementary Material 5 [file 12885_2023_11509_MOESM5_ESM.doc]
