# Supplementary material for: Toll-like receptor 9 (-1237 T/C, -1486 T/C) and the risk of gastric cancer: a meta-analysis of genetic association studies
Source: BMC Cancer. 2023 Oct 24;23:1027. doi: 10.1186/s12885-023-11509-7 (PMC10594725; doi:10.1186/s12885-023-11509-7)
Supplement: Supplementary file 2 — Supplementary Material 2 [file 12885_2023_11509_MOESM2_ESM.doc]

**Additional File 3. The excluded ten studies**

|  | Study, year | Main reason | Reference |
| --- | --- | --- | --- |
| 1 | Loganathan 2017 | Case- control design of *H.Pylori* infection, but not with GC. | Loganathan R, Nazeer M, Goda V, et al. Genetic variants of TLR4 and TLR9 are risk factors for chronic *Helicobacter pylori* infection in South Indian Tamils*. Hum Immunol*. 2017;78(2):216-220 |
| 2 | Upcinskas 2011 | not with targeted SNP | Kupcinskas J, Wex T, Bornschein J. *et al.* Lack of association between gene polymorphisms of Angiotensin converting enzyme, Nod-like receptor 1, Toll-like receptor 4, FAS/FASL and the presence of *Helicobacter pylori*-induced premalignant gastric lesions and gastric cancer in Caucasians. BMC Med Genet 2011; 12, 112 |
| 3 | Balbaloglu, 2017 | not with GC. | Balbaloglu O, Sabah Ozcan S, Korkmaz, M. et al Promoter polymorphism (T-1486C) of TLR-9 gene is associated with knee osteoarthritis in a Turkish population. J. Orthop. Res.2017, 35: 2484-2489. |
| 4 | Santini, 2008 | not with targeted SNP | Santini D, Angeletti S, Ruzzo A, et al Toll-like receptor 4 Asp299Gly and Thr399Ile polymorphisms in gastric cancer of intestinal and diffuse histotypes. Clin Exp Immunol. 2008;154(3):360-4 |
| 5 | Chen, 2018 | not with targeted SNP | Chen G, Xu M, Chen J, et al. Clinicopathological features and increased expression of toll-like receptor 4 of gastric cardia cancer in a high-risk Chinese population. J Immunol Res. 2018; 7132868. |
| 6 | Zhou, 2014 | A review and not with targeted SNP | Zhou Q, Wang C, Wang X, et al/. Association between TLR4 (+896A/G and +1196C/T) polymorphisms and gastric cancer risk: an updated meta-analysis. PLoS One. 2014;9(10):e109605. |
| 7 | Huang, 2022 | not with targeted GC | Huang Q, Wang CC, Liu YG, et al Clinical relevance of genetic polymorphisms in WNT signaling pathway (*SFRP1*, *WNT3A*, *CTNNB1, WIF-1*, *DKK-1*, *LRP5*, *LRP6*) on pulmonary tuberculosis in a Chinese population. Front Immunol. 2022;13:1011700. |
| 8 | Eskuri, 2023 | No control group, a cohort study. | Eskuri M, Kemi N, Helminen O, et al. Toll-like receptors 3, 7, 8, and 9 in gastric cancer. APMIS. 2023; 131(2):92-99 |
| 9 | Kasurinen,2019 | Not a genetic association study | Kasurinen A, Hagström J, Laitinen A, et al Evaluation of toll-like receptors as prognostic biomarkers in gastric cancer: high tissue TLR5 predicts a better outcome. Sci Rep. 2019;9(1):12553. doi: 10.1038/s41598-019-49111-2. |
| 10 | Tang, 2022 | Not a genetic association study | Tang K, McLeod L, Livis T, et al Toll-like receptor 9 promotes initiation of gastric tumorigenesis by augmenting inflammation and cellular proliferation. Cell Mol Gastroenterol Hepatol. 2022;14:567-586. |

GC: gastric cancer.
